# Relationship between Primary and Secondary Dental Care in Public Health Services in Brazil

**DOI:** 10.1371/journal.pone.0164986

**Published:** 2016-10-18

**Authors:** Renata Castro Martins, Clarice Magalhães Rodrigues dos Reis, Antonio Thomaz Gonzaga da Matta Machado, João Henrique Lara do Amaral, Marcos Azeredo Furquim Werneck, Mauro Henrique Nogueira Guimarães de Abreu

**Affiliations:** 1 Department of Social and Preventive Dentistry, Faculty of Dentistry, Universidade Federal de Minas Gerais, Belo Horizonte, Minas Gerais, Brazil; 2 Postgraduate Program in Dentistry, Faculty of Dentistry, Universidade Federal de Minas Gerais, Belo Horizonte, Minas Gerais, Brazil; 3 Department of Preventive and Social Medicine, Faculty of Medicine, Universidade Federal de Minas Gerais, Belo Horizonte, Minas Gerais, Brazil; University of North Carolina at Chapel Hill, UNITED STATES

## Abstract

This cross-sectional study evaluated the relationship between primary and secondary oral health care in Brazil. For this purpose, data from the National Program for Improving Access and Quality of Primary Care were used. Dentists from 12,403 oral health teams (OHTs) answered a structured questionnaire in 2012. The data were analyzed descriptively and by cluster analysis. Of the 12,387 (99.9%) OHTs that answered all the questions, 62.2% reported the existence of Dental Specialties Centers (DSCs) to which they could refer patients. The specialties with the highest frequencies were endodontics (68.4%), minor oral surgery (65.8%), periodontics (63.0%), radiology (46.8%), oral medicine (40.2%), orthodontics (20.5%) and implantology (6.2%). In all percentiles, the shortest wait time for secondary care was for radiology, followed by oral medicine and the other specialties. In the 50th percentile, the wait for endodontics, periodontics, minor oral surgery and orthodontics was 30 days, while for implantology, the wait was 60 days. Finally, in the 75th percentile, the wait for endodontics, orthodontics and implantology was 90 days or more. Two clusters, with different frequencies of OHT access to specialties, were identified. Cluster 1 (n = 7,913) included the OHTs with lower frequencies in all specialties except orthodontics and implantology compared with Cluster 2 (n = 4,474). Of the Brazilian regions, the South and Southeast regions had the highest frequencies for Cluster 2, with better rates for the relationship between primary and secondary care. This study suggests certain difficulties in the relationship between primary and secondary care in specific specialties in oral health, with a great number of OHTs with limited access to DSCs, in addition to different performance in terms of OHT access to DSCs across Brazilian regions.

## Introduction

The integration of primary and secondary care is a relevant public health issue in both developed [1–2] and underdeveloped countries [3–4].

Many developed countries are considering integrated care to help to deliver more cost-effective, high-quality care. Various examples of successful integration of primary and secondary care have been reported in the literature, and these examples have all focused on a combination of several, if not all, of the following elements: joint planning, integrated information communication technology, change management, shared clinical priorities, aligned incentives, population-focused care, measurement, professional development, community/patient engagement and innovation. There appears to be agreement that multiple elements are required to ensure successful and sustained integration efforts. Additionally, while no single model fits all systems, these elements provide a focus for setting up integration initiatives, which need to be flexible for adaptation to local conditions and settings [1–2].

The interface between primary and secondary dental care displays three key features: interdependence, integration and complexity. Specifically, primary and secondary care providers are dependent on each other, as primary care is the main source of referrals to secondary care once patients need specialized treatment. However, patients need to return to primary care for routine, maintenance care. Cooperation and good communication are essential for a successful interface, with both sides needing to be clear about what the other is requesting or proposing, particularly when the treatment requires them to coordinate their efforts. The dental interface is also complex: secondary care services are subject to a range of influences that drive the referral rate and are affected by number of options for managing increased referrals. Many factors can be drivers or inhibitors of referrals to secondary dental care, such as wait times, specialist skills, patient demand, health policy, prioritizing decisions or protocols, clinical guidance, access problems, and treatment within the primary care setting [5–7].

In Brazil, health was recognized as a right of all citizens and a duty of the state in 1988, with the establishment of the Brazilian Health System (in Portuguese, *Sistema Único de Saúde*, or SUS). The Brazilian Health System brought a new political and organizational formulation to the reorganization of services and health programs, establishing promotional activities as well as ensuring the protection and recovery of health [8–9]. The financing of the Brazilian Health System is provided by the three levels of government, namely, federal, state and local, as required by the Federal Constitution [10], which established the resources available to cover the costs of actions and public health services. Additionally, the Family Health Program, created in 1994, prioritizes primary health care (PHC) for the population and the epidemiological needs of the country. In 2000, oral health teams (OHTs) were included among the Family Health Program teams, restructuring the PHC model and bringing a new perspective to oral health action planning in the public sector; in this sector, the fundamental basis of action is territorial and the focus is on the social determinants of health and the epidemiological needs of the population [8]. In 2004, National Oral Health Policy guidelines, called “*Brasil Sorridente*”, were created, emphasizing the need to increase access to oral health care with a full view of health and disease processes, including promotion, protection and recovery of health and incorporating procedures and services of high and medium complexity as well as intersectoral activities [9, 11–13]. This new policy was responsible for expanding and qualifying services in oral health, increasing the resolution of actions, providing dental care within the primary care network and deploying Dental Specialties Centers (DSCs) [12–13]. These DSCs are referral centers for primary care that are integrated into the processes of local and regional planning and that proffer minimal periodontics, endodontics, care for patients with special needs, oral diagnosis and minor oral surgery, providing procedures that are specialized, complex, and complementary to primary care [9,12–14]. Prosthetics, however, are not included as a specialty among DSCs; these are instead offered in primary care. According to the last national survey [15], a high percentage of adults and elderly individuals require prostheses. However, Reis et al. [16] observed that although 50.5% of OHTs identify people in need of dentures, dentures and other prostheses are rarely offered in PHC.

The Brazilian territory is divided into five major regions: South, Southeast, Midwest, Northeast and North. These divisions reflect the diversity of resources and conditions available for economic and social development [6,8,15]. The South region is the smallest in terms of land area; the third largest in terms of the number of inhabitants; and the second in terms of population density, behind the Southeast. This region is known for its cities with a model quality of life. Meanwhile, the Southeast is the most populous, industrialized and technologically advanced region of Brazil. This region has the highest population density and the highest concentration of household income in the country, and it is a major contributor to Brazil’s gross domestic product. However, the Midwest region is economically the fastest growing region of Brazil. This region has the second largest area, following the North. The Northeast is one of the poorest regions of Brazil and is the second largest in Brazil in terms of population, following the Southeast. Advances in this region have not yet translated into improvements in the population’s quality of life, especially regarding the availability of infrastructure, such as sanitation, in various locations. Finally, the North region has the largest land area among the regions of Brazil. However, despite being the largest region, the North has one of the smallest populations in absolute terms and one of the lowest population densities in the country. Following the Northeast, this region also faces the greatest social and economic challenges among the regions of the country [17,18].

Although all Brazilian citizens can use the public services, use is in fact associated with family income, how this income is divided within households, the need for treatment presented by the individual and the organization of the existing oral health service infrastructure in the municipality [19]. According to Pinto et al. [20], 51.8% of the adult population in Brazil uses public services, 37.1% uses liberal private services and 11.2% uses health plans. The population attended by the public services is socioeconomically less privileged and has a greater need for treatment [19–21]. The greatest chance of using public health care services is related to being black, having a greater number of household residents, having a lower family income, being an inhabitant of a small town, and having a larger number of teeth needing treatment [21]. The public services are the most widely used services in all regions, as is the option of going to the dentist for prevention or treatment. The use of public services is higher in the North (65.4%) compared with the Southeast (43.2%), the South (41.3%) and Brazil overall (46.3%). For adults in all regions, the reason most cited for their last visit to the dentist is treatment, while for the elderly, there is a significant difference between regions. The North region presents a higher proportion of elderly (46.2%) who use public dental services for dental extraction, and 24.9% of elderly people use the same services for treatment. In the South region, the situation is reversed (22.9% and 38.3%, respectively) [15]. Inequality in use of public dental services is linked to education and the organization of public services. Local governmental management with better governance, design and capacity has better-organized public dental services and is associated with less use of private services, reinforcing the idea that these subsystems are not complementary, but rather compete with each other [19,22].

However, within PHC, oral health still has many challenges to overcome, including precarious labor relations, difficulty integrating primary and secondary oral health care, excess demand, difficulty in planning and evaluating actions and a need to improve the quality of care provided to the population [7,23–24]. In this context, assessment of the quality of primary care services in Brazil is appealing. In 2011, the National Program for Improving Access and Quality of Primary Care (PMAQ-AB) was created. The main challenge of the PMAQ-AB is to establish a culture of evaluation among PHC professionals and managers who monitor and evaluate processes and results. To this end, the program evaluates the performance of teams and provides financial incentives based on the results [25].

Given the importance of assessing the interface between primary and secondary dental care, the aim of the present study was to describe the state of secondary dental care services among OHTs in Brazil.

## Materials and Methods

This study was conducted in full accordance with the ethical principles of the Declaration of Helsinki and additional requirements. The study was independently reviewed and approved by the Human Research Ethics Committee of the *Universidade Federal de Minas Gerais*, Brazil (Protocol Number 31525514.9.0000.5149).

This cross-sectional descriptive study used data from a national survey on PHC teams conducted by the Brazilian Ministry of Health through the PMAQ-AB. The goal of this project was to improve access to and the quality of PHC by technically and economically supporting PHC teams. This goal implies an accreditation process based on the results of an external evaluation and an analysis of health outcomes achieved by each PHC team. The PMAQ-AB is based on the classic quality-of-care framework proposed by Donabedian, in which quality is evaluated using structure, process and outcome parameters [26–27].

The study population consisted of dentists in Brazil working on OHTs who completed a PMAQ-AB survey regarding oral health care. In 2012, a total of 12,403 OHTs (response rate of 85.01%) completed the survey questions. The questions were based on the principles of PHC that should be incorporated by the OHTs, such as access to care and health service organizations as well as continuity of care. The sampling details can be found in a previous study [16].

The Brazilian Ministry of Health partnered with academic institutions to develop the questionnaire and administer the survey, including the selection and training of interviewers who were to administer the survey across the country. Data were specifically collected in face-to-face interviews at a PHC unit through a structured questionnaire in 2012 (S1 File). The dentists participating in this project were volunteers and could refuse to answer the questionnaire. The questionnaire consisted of mostly dichotomous questions, although each question included an option for “no answer/do not know” [16].

This study focused on reference centers for dental specialties. The following questions about centers of reference for dental specialties were asked: “Are there Dental Specialties Centers to which your team can refer patients?” and “For which specialties does the city have a reference center (endodontics, periodontics, minor oral surgery, oral medicine, orthodontics, implantology, radiology, other)?” Questions were also asked about wait times for dental specialties, including “After being requested by the primary care professional, how long, on average, should the user expect to wait for treatment for the following specialties (endodontics, periodontics, minor oral surgery, oral medicine, orthodontics, implantology, radiology, other)?”

Data for the Brazilian population and the number of DSCs by macro-region were collected from the Brazilian Institute of Geography and Statistics [28] and the Support Strategic Management Room [29], respectively.

Descriptive statistics were analyzed and a cluster analysis was performed using the IBM Statistical Package for Social Sciences (SPSS), version 19.0 (IBM SPSS Statistics for Windows, Armonk, NY). Confidence intervals were not calculated because this was a census study. Although the Brazilian Health System may have geographical differences in the quality of its services, for the variable that we evaluated, no previous results have shown any differences. Therefore, we decided to use the multivariate agglomerative hierarchy technique based on the farthest neighbor with squared Euclidian distances for the cluster analysis because it is an exploratory data analysis tool for organizing observed data (in our case, from OHTs) into groups (clusters), is based on combinations of independent variables (in our case, references to DSCs and the access of OHTs to each specialty), and maximizes the similarity of cases within each cluster while maximizing the dissimilarity between groups. This multivariate analysis also creates new groupings without any preconceived notion of what clusters may arise. The clustering was based on the following variables: the frequency of OHT access to DSCs and the access of OHTs to each specialty. This data reduction made the management of subgroups easier. A major issue in cluster analysis is how to choose the final number of groups, and there is no exact answer to this question. Here, three sets of clusters (two to four) were formed from the 12,387 OHTs, and the choice of two clusters was based on improved understanding of the phenomenon (the characteristics of referrals from primary care to secondary care). Dendogram use also helped with visual inspection of the distances between clusters [30]. One OHT was not placed in any cluster.

## Results

Among 12,403 OHTs, a total of 12,388 (99.89%) were considered in the descriptive analysis, as they answered all the questions about the relationship between primary and secondary care in oral health in the Brazilian public sector (Table 1).

**Table 1:** Descriptive analysis of the existence of a DSC to which to refer patients according to the PMAQ-AB data, 2012.

Variable (n = 12,388)	Frequency (%)*
Is there a Dental Specialties Center to which your team can refer patients?	7,703 (62.2)
Does the city have endodontics at the reference center?	8,468 (68.4)
Does the city have periodontics at the reference center?	7,807 (63.0)
Does the city have minor oral surgery at the reference center?	8,152 (65.8)
Does the city have oral medicine at the reference center?	4,977 (40.2)
Does the city have orthodontics at the reference center?	2,537 (20.5)
Does the city have implantology at the reference center?	773 (6.2)
Does the city have radiology at the reference center?	5,805 (46.8)
Other	2,568 (20.7)

*considering only the valid results.

Overall, 62.2% of OHTs reported the existence of a DSC to which to refer patients. The most common specialties referenced by the OHTs were endodontics (68.4%), followed by minor oral surgery (65.8%) and periodontics (63.0%). Radiology and oral medicine were cited by 46.8% and 40.2% of the evaluated OHTs, respectively, while orthodontics and implantology were the least cited (20.5% and 6.2%, respectively).

Table 2 shows the time that a patient must wait for secondary care treatment after being referred by the primary care professional. As these data presented a non-normal distribution, percentiles were used. The range for each specialty was very high. In all percentiles, the shortest wait time for secondary care was for radiology, followed by oral medicine and the other specialties. In the 50th percentile, the wait for endodontics, periodontics, minor oral surgery and orthodontics was 30 days, while for implantology, it was 60 days. Finally, in the 75th percentile, the wait for endodontics, orthodontics and implantology was 90 days or more.

**Table 2:** Percentiles for the patient wait time, in days, for secondary care treatment after being requested by the primary care professional according to the PMAQ-AB data, 2012.

Specialty (n = 12,388)	Percentile*		
	25%	50%	75%
Endodontics	20.00	30.00	99.00
Periodontics	14.00	30.00	50.00
Minor oral surgery	12.25	30.00	45.00
Oral medicine	7.00	15.00	30.00
Orthodontics	15.00	30.00	99.00
Implantology	20.00	60.00	90.00
Radiology	0.00	2.00	8.00
Other	7.00	15.00	30.00

*considering only the valid results.

Three sets of clusters (two to four) were formed from the 12,387 OHTs, and the choice of two clusters was based on improved understanding of the phenomenon (the characteristics of referrals from primary care to secondary care). Table 3 shows the existence of DSCs to which the OHTs could send patients. In Cluster 2 (n = 4,474), 99.1% of the OHTs had access to a DSC, while in Cluster 1 (n = 7,913), only 41.3% of the OHTs did.

**Table 3:** Existence of a DSC to which the OHT can refer patients from primary to secondary dental care by cluster, Brazil, 2012.

Variable	Cluster 1* (n = 7,913)	Cluster 2* (n = 4,474)
Is there a Dental Specialties Center to which your team can refer patients?	41.3%	99.1%
Does the city have endodontics at the reference center?	54.9%	92.2%
Does the city have periodontics at the reference center?	50.1%	85.9%
Does the city have minor oral surgery at the reference center?	53.3%	87.9%
Does the city have oral medicine at the reference center?	27.4%	62.7%
Does the city have orthodontics at the reference center?	29.3%	4.8%
Does the city have implantology at the reference center?	9.6%	0.3%
Does the city have radiology at the reference center?	40.7%	57.7%
Other	14.1%	32,5%

*considering only the valid results.

Table 4 shows the distribution of DSCs in Brazil, considering the population of each macro-region. It can be observed that in 2012, Brazil had a population of 190,755,799 [28], with a total of 994 DSCs distributed across its five macro-regions [29]. The rate of DSCs per 1,000,000 inhabitants varied from 4.10 in the North region to 6.91 in the Northeast. There was an unequal distribution of clusters across the macro-regions. In particular, the South and Southeast regions had the highest proportions of Cluster 2, with better performance in terms of OHT access to DSCs.

**Table 4:** Distribution of Brazilian macro-regions, DSCs and population by cluster, Brazil, 2012.

Brazilianmacro-region	DSC(n)	Population	DSCs/1,000,000inhabitants(rate)	Cluster 1*(n = 7,780)	Cluster 2*(n = 4,362)
South	115	27,386,891	4.20	58.2%	41.8%
Southeast	330	80,364,410	4.11	62.2%	37.8%
Midwest	67	14,058,094	4.77	68.4%	31.6%
Northeast	367	53,081,950	6.91	66.3%	33.7%
North	65	15,864,454	4.10	72.6%	27.4%
Total	944	190,755,799	4.95		

*considering only the valid results.

## Discussion

This large survey showed that in Brazil, there is a structure of secondary oral health care in public services, with differences in the frequency and geographical localization of specialties.

Since the DSCs were created by the Brazilian Ministry of Health in 2004, procedures in endodontics, periodontics, minor oral surgery, oral medicine and care for patients with special needs have been offered at these centers [8–9,12,14]. As oral health policy is recent, a large proportion of OHTs unfortunately still do not have access to a DSC.

In the current study, the specialties that were most frequently cited by OHTs as presently available secondary care services were endodontics, periodontics and minor oral surgery. Oral health problems, such as dental caries and periodontal disease, are prevalent in adults and are considered a major public health problem [6,31]. The frequency of decay often necessitates endodontic treatment, increasing the demand for this specialty. Severe periodontal disease, however, requires special treatment and often needs more complex surgery [6,32]. Thus, these three specialties were implemented first at the DSCs.

The fact that oral medicine was not often reported by OHTs as a secondary care service may be because of a lack of professionals in this specialty and difficulty in hiring in the Brazilian Health System, which limits the services offered [7]. The lack of these services is serious because early diagnosis of malignant lesions is essential for the integrality of oral health care [4,7]. The integration of primary and secondary health care particularly enables the diagnosis of potentially malignant lesions and squamous cell carcinoma and establishes a diagnostic network. However, most primary care dentists reported feeling incapable of performing biopsies, most likely because of anxiety about oral cancer. In fact, many dentists had never performed a biopsy. The inability of most primary care dentists to identify potentially malignant lesions and perform biopsies is a weakness in the diagnostic process [4]. Reis et al. [16] found that OHTs adhere to several of the principles of primary care organizations, but the teams perform fewer procedures related to oral cancer treatment and to rehabilitation with complete dentures. In addition to the failure to identify oral lesions early, other factors are associated with the delayed arrival of patients with oral cancer to the health sector, such as a lack of multidisciplinary work, disrupted network care given the poor quality of communication between professionals at different levels of care [33], and a lack of diagnostics for oral lesions provided by dentists in their clinical practice.

Orthodontics and implantology were included at DSCs in 2011 [34]. As these are recently included specialties, they still offer few treatment options. These specialties were specifically incorporated into DSCs after a Brazilian oral health survey [15] showed a high prevalence of malocclusion and tooth loss in the population. The lack of dental specialty services is partly a reflection of the limited resources available to certain regions, but other factors, such as the planning and organization of services, are also defining [5–7]. Lino et al. [35] analyzed secondary oral health care in Minas Gerais state, Brazil, and observed a significant number of specialized procedures being performed in primary care settings, which is not in accordance with the Brazilian National Oral Health Policy [9–13]. To eliminate regional differences in access to dental specialty services, the federal and state governments must improve access to services and training of professionals and must encourage more resolutive primary care to reduce the demand for secondary care, in addition to improving system management. These steps are politically and economically feasible, but at the moment, the Brazilian economic crisis poses a challenge.

Brazil is a country of continental dimensions that is divided into five geographical regions with differing demographic, economic, cultural, and health conditions and widespread internal inequalities [18]. This heterogeneity is reflected in health services, which have different organizational structures, and in the populations, which differ in their oral health status. The variation in wait times cited for different specialties is probably due to differences in the work processes of each specialty, which may also explain the range within each specialty. In addition, other factors can influence referrals to secondary dental care, such as inadequate resources; poor management; allocation of resources among medical, dental and social programs within regions; population demographics; the number of professionals available; specialist skill; patient demand; health policy; prioritizing decisions or protocols; clinical guidance; access problems; and treatment within the primary care setting [5–7]. The wait time is a problem of health management and can lead to no-shows by patients [36]. In the present study, radiology and oral medicine had the shortest wait times, probably because these specialties can solve dental problems faster than the other specialties can. In contrast, orthodontics, implantology and endodontics presented longer wait times, in days, in the 75th percentile. For orthodontics and implantology, the increased wait times are probably due to these specialties’ status as recently deployed services for which few professionals are presently available, while for endodontics, the increased wait time may be due to great patient demand for this specialty. Other factors may also be related to wait times for secondary care treatment after this treatment is requested by the primary care professional, and these factors need to be explored. Such factors may reflect differences between cities with and without DSCs, such as the distance to the nearest DSC, the number of quotes and the financial and logistical resources available to send patients to a secondary care facility, in addition to patient socioeconomic status and cultural aspects.

In the present study, the OHTs in Cluster 2 had more access to DSCs and to the endodontics, periodontics, minor oral surgery, and oral medicine specialties. This group of OHTs had reference centers for secondary care that were consistent with the Brazilian National Oral Health Policy [9–13]. However, the other group of OHTs (Cluster 1) had more limited access to DSCs and to traditional specialties (endodontics, periodontics, minor oral surgery and oral medicine). It is necessary to investigate the OHTs that refer patients to specialties that are not traditional and that do no not represent Brazilian epidemiological demand, such as orthodontics and implantology [15], but that were recently deployed in the Brazilian Health System; perhaps these reference centers are dental schools that offer such specialized treatments.

Brazil has a vast territory with considerable social, economic and epidemiological differences [8,15,37]. In the Brazilian population, dental treatment needs related to primary care and secondary care are strongly associated with individual socioeconomic status, and especially income and education. A core principle of the Brazilian Health System is that the distribution of health services must equitable, which implies positive discrimination in terms of priorities [37]. The capacity to access dental services is still strongly modulated by socioeconomic status, however, as dental services utilization is often associated with socioeconomic variables [38]. It seems that this inequality decreases with the existence of secondary referral centers for OHTs. In the current study, however, the existence of more DSCs in the Northeast did not result in a higher frequency of OHTs in Cluster 2, which had better performance in terms of access to DSCs. Previous studies have shown that cities with DSCs are characterized by better social conditions [35,39]. Since the creation of the DSCs, the quantity throughout Brazil has been steadily growing. However, there is still a pattern of concentration in the Northeast and Southeast [40–41], which are the most populous regions of Brazil. Specifically, the Northeast region has the largest number of DSCs and family health teams and presents unfavorable social indicators [20]. The equity principle has guided the dissemination of DSCs because the Northeast has a high concentration of large deficiencies in oral health and unfavorable social indicators [36]. Given that health service organizations can be influenced by socioeconomic and demographic variables, it is understandable that the Northeast region presented a higher frequency of OHTs in Cluster 1 than the Southeast and South regions did in the present study. Reis et al. [16] found that the Northeast region was composed of OHTs with the lowest proportion of oral health care actions related to oral cancer treatment and construction of dental prostheses/dentures. It is also important to emphasize that there is a tendency of professionals to be concentrated in more developed regions, in contrast to their absence in less developed regions. A private system exists in Brazil, and people can access it depending on their economic status. In fact, the Brazilian adult population often uses private or supplementary services [15]. However, it is not common for the public sector to purchase private services, and patients in regions with a paucity of specialty services frequently cannot pay for private services. Social conditions and access to health services influence tooth loss, as in many cases, the population without access to specialty services resorts to extraction [42].

The present study suggests certain difficulties in the relationship between primary and secondary care in specific specialties in oral health, with a large number of OHTs having limited access to DCS, in addition to different performance in terms of OHT access to DSCs across Brazilian regions. Our results should not be extrapolated to the entire Brazilian Health System, considering the limited proportion of OHTs that was evaluated in this survey. The scant presence of similar studies regarding Brazil in the literature, especially regarding this specific topic, is another limitation. The data for computing the “wait time” were obtained by interviewing the OHTs, and we also acknowledge the limitations of this data, considering that information from the reference centers (to which we do not have access) as well as from patients (again, to which we do not have access) could also improve comprehension of this topic.

The description of the relationship between primary and secondary care may contribute to planning and management in health services. Weak points in the health network must be identified to ensure that public policies are implemented to advance the health care of the relevant populations.

## Supporting Information

S1 File Dataset. Modulo IIforPLOSONE.sav.
(SAV)

## References

[1] Armitage GD, Suter E, Oelke ND, Addair CE. Health systems integration: state of evidence. Int J Integr Care. 2009 9(17):e82. 19590762 10.5334/ijic.316 PMC2707589

[2] Nicholson C, Jackson, Marley J. A governance model for integrated primary/ secondary care for the health-reforming first world—results of a systematic review. BMC Health Serv Res. 2013 13:528 10.1186/1472-6963-13-528 24359610 PMC4234138

[3] Dudley L, Garner P. Strategies for integrating primary health services in low- and middle-income countries at the point of delivery. Cochrane Libr. 2011 6(7):1–73. 10.1002/14651858.CD003318.pub3 PMC6703668 21735392

[4] Souza FB, Silva MRF, Fernandes CP, Silva PGB, Alves APNA. Oral cancer from a heatlh promotion perspective: experience of a diagnosis network in Ceara. Braz Oral Res. 2014 28(Spec Iss 1):1:8. 10.1590/1807-3107BOR-2014.vol28.0018 24964281

[5] Morris AJ, Burke FJT. Primary and secondary dental care: the nature of the interface. Practice Health Police. Br Dent J. 2001 191(12):660–4. 10.1038/sj.bdj.4801262a 11792111

[6] Kandelman D, Arpin S, Baez RJ, Baehni PC, Petersen PE. Oral health care systems in developing and developed countries. Periodontol 2000. 2012 60(1):98–109. 10.1111/j.1600-0757.2011.00427.x 22909109

[7] Godoi H, Mello ALSF, Caetano JC. An oral health care network organized by large municipalities in Santa Catarina state, Brazil [portuguese]. Cad Saude Publica. 2014 30(2):318–32. 10.1590/0102-311X00084513 24627060

[8] Junqueira SR, Pannuti CM, Rode SM. Oral health in Brazil—Part I: public oral health policies. Braz Oral Res. 2008 22(Spec Iss 1):8–17. 19838546 10.1590/s1806-83242008000500003

[9] Pucca GA Jr, Costa JFR, Chagas LD, Sivestre RM. Oral health policies in Brazil. Braz Oral Res. 2009 23(Spec Iss 1):9–16. 19838553 10.1590/s1806-83242009000500003

[10] Brazil. Presidency of the Republic. Civil House. Federal Republic of Constitution of 1988 of Brazil [portuguese]. 1988. [cited 2016 Jul 02]. Available in http://www.planalto.gov.br/ccivil_03/constituicao/constituicaocompilado.htm http://189.28.128.100/dab/docs/portaldab/publicacoes/manual_instrutivo_PMAQ_AB2013.pdf

[11] Levcovittz E, Lima LD, Machado CV. Health policy in the 1990s: inter-governmental relations and the Basic Operational Norms [portuguese]. Cien Saude Colet. 2001 6(2):269–91.

[12] Brazil. Ministry of Health. Guidelines of the National Oral Health Policy [portuguese]. 2004a. [cited 2015 Sep 10]. Available: http://www.saude.gov.br/bucal.

[13] Brazil. Ministry of Health. Health Programs. Brasil Sorridente [portuguese]. 2004b. [cited 2015 Sep 10]. Available in http://www.saude.gov.br.

[14] Pedrazzi V, Dias KRHC, Rode SM. Oral health in Brazil—Part II: dental specialty centers (DSC). Braz Oral Res. 2008 22(Spec Iss 1).18–22. 10.1590/s1806-83242008000500004 19838547

[15] Brazil. ProjectoSBBrasil 2010: National Oral Health Survey—Principal Findings [portuguese]. 2011. [cited 2015 Sep 10]. Available in: http://dab.saude.gov.br/CNSB/sbbrasil/arquivos/projeto_sb2010_relatorio_final.pdf

[16] Reis CMR, Matta-Machado ATG, Amaral JHL, Werneck MAF, Abreu MHNG. Describing the primary care actions of oral health teams in Brazil. Int J Environ Res Public Health. 2015 12(1):667–78. 10.3390/ijerph120100667 25588158 PMC4306885

[17] Griesse MA. The geographic, political, and economic context for corporate social responsibility in Brazil. Journal of Business Ethics. 2007 73:21–37.

[18] Paim J, Travassos C, Almeida C, Bahia L, Macinko J. The Brazilian health system: history, advances, and challenges. The Lancet. 2011 377:1778–1797. 10.1016/S0140-6736(11)60054-8 21561655

[19] Pinto RS, Roncalli AG, Abreu MHNG, Vargas AMD. Use of public oral health services by the adult population: a multilevel analysis. Plos One. 2016 Available in: http://journals.plos.org/plosone/article?id=10.1371/journal.pone.0145149 10.1371/journal.pone.0145149 PMC4701482 26730714

[20] Pinto RS, Matos DL, Loyola Filho AI. Characteristics associated with the use of dental services by the adult Brazilian population [portuguese]. Cien Saude Colet. 2012 17(2):531–544. 22267047 10.1590/s1413-81232012000200026

[21] Pinto RS, Abreu MHNG, Vargas AM. Comparing adult users of public and private dental services in the state of Minas Gerais, Brazil. BMC Oral Health. 2014 14:100 10.1186/1472-6831-14-100 25099268 PMC4130879

[22] Soares FF, Chaves SCL, Cangussu MCT. Local government and public dental health services: an analysis of inequality in use [portuguese]. Cad Saúde Pública. 2015 31(3):586–596. 25859725 10.1590/0102-311x00077214

[23] Santos AM, Assis MMA. From fragmentation to integrality: constructing and reconstructing the practice of buccal health in the Alagoinhas (BA) Family Health Program [portuguese]. Cien Saude Colet. 2006 11(1):53–61.

[24] Anjos F, Mestriner SF, Bulgarelli AF, Pinto IC, Mestriner-Junior W. Brazilian oral health crew: advances and challenges [portuguese]. Cien Cuid Saude. 2011 10(3):601–7.

[25] Brazil. Ministry of Health. Health closer to you—Access and Quality—Programme for Improving Access and Quality of Primary Care (PMAQ-AB)—Instruction manual [portuguese]. 2012. [cited 2015 Sep 10]. Available in http://189.28.128.100/dab/docs/portaldab/publicacoes/manual_instrutivo_PMAQ_AB2013.pdf.

[26] Donabedian A. Evaluating the quality of medical care. 1966. Milbank Q. 2005 83(4):691–729. 16279964 10.1111/j.1468-0009.2005.00397.x PMC2690293

[27] Voinea-Griffin A, Fellows JL, Rindal DB, Barasch A, Gilbert GH, Safford MM. Pay for performance: will dentistry follow? BMC Oral Health. 2010 10:9 10.1186/1472-6831-10-9 20423526 PMC2880362

[28] Brazil. Brazilian Institute of Geography and Statistics. Census 2010 [portuguese]. 2010 [cited 2015 Sep 10]. Available in: http://censo2010.ibge.gov.br/.

[29] Brazil. Ministry of Health. Support Strategic Management Room. Brasil Sorridente/ Dental Specialties Centers—DSC/Dental prosthesis [portuguese]. [cited 2015 Sep 10]. Available in: http://189.28.128.178/sage/.

[30] Johnson RA, Wichem DW. Applied multivariate statistical analysis.6. Pearson Prentice Hall: Upper Saddle River, NJ; 2007.

[31] Petersen EP, Bourgeoi SD, Ogawa H, Estupinan-Day S, Ndiaye C. The global burden of oral diseases and risk to oral health. Bull World Health Org. 2005 83(9):661–669. 16211157 PMC2626328

[32] Kim J, Amar S. Periodontal disease and systemic conditions: a bidirectional relationship. Odontology. 2006 94(1):10–21. 10.1007/s10266-006-0060-6 16998613 PMC2443711

[33] Lombardo EM, Cunha AR, Carrad VC, Bavaresco CS. Delayed referrals of oral cancer patients: the perception of dental surgeons [portuguese]. Cien Saude Colet. 2014 19(4); 1223–32. 24820605 10.1590/1413-81232014194.00942013

[34] Brazil. Ministry of Health. Health Care Secretary. General Coordination of Oral Health. Ordinance 718/SAS [portuguese]. [cited 2015 Sep 10]. Available in: http://189.28.128.100/dab/docs/geral/nota_portaria718_sas4.pdf.

[35] Lino AP, Werneck MAF, Lucas SD, Abreu MHNG. Analysis of secondary care in oral health in the State of Minas Gerais, Brazil [portuguese]. Cien Saude Colet. 2013 19(9);3879–88. 10.1590/1413-81232014199.12192013 25184593

[36] Machado AT, Werneck MAF, Lucas SD, Abreu MHNG. Who did not appear? First dental visit absences in secondary care in a major Brazilian city: a cross-sectional study. Cien Saude Colet. 2015 20(1):289–98. 10.1590/1413-81232014201.01012014 25650623

[37] Roncalli AG, Tsakos G, Sheiham A, Souza GC, Watt RG. Social determinants of dental treatment needs in Brazilian adults. BMC Public Health. 2014 14:1097 10.1186/1471-2458-14-1097 25339315 PMC4287338

[38] Peres KG, Peres MA, Boing AF, Bertoldi AD, Bastos JL, Barros AJ. Reduction of social inequalities in utilization of dental care in Brazil from 1998 to 2008. Rev Saude Publica. 2012 46(2):250–58. 22437856 10.1590/s0034-89102012000200007

[39] Celeste RK, Moura FRR, Santos CP, Tovo MF. Analysis of outpatient care in Brazilian municipalities with and without specialized dental clinics, 2010 [portuguese]. Cad Saude Publica. 2014 30(3);511–21. 24714941 10.1590/0102-311x00011913

[40] Goes PSA, Figueiredo N, Neves JC, Silveira FMM, Costa JFR, Pucca GA Jr, Rosales MS. Evaluation of secondary care in oral health: a study of specialty clinics in Brazil [portuguese]. Cad Saúde Pública. 2012 28:S81–S89. 22714971 10.1590/s0102-311x2012001300009

[41] Cortelazzi KL, Balbino EC, Guerra LM, Vazquez FL, Bulgareli JV, Ambrosano GMB, Pereira AC, Mialhe FL. Variables associated with the performance of Centers for Dental Specialties in Brazil. Rev Bras Epidemiol. 2014 17(4): 978–988. 25388496 10.1590/1809-4503201400040015

[42] Cunha MAGM, Lino PA, Santos TR, Vasconcelos M, Lucas SD, Abreu MHNG. A 15 year time series study of tooth extraction in Brazil. Medicine. 2015 94(47): e1924 10.1097/MD.0000000000001924 26632688 PMC5058957

